# Evaluation of a Witch Hazel Extract for the Potential Prebiotic and Protective Effect on Select *Lactiplantibacillus plantarum* (Prev. *Lactobacillus plantarum*) Strains

**DOI:** 10.3389/fnut.2022.874666

**Published:** 2022-04-29

**Authors:** Morgan Failla, Jungyun Lee, Reuven Rasooly, Emmanouil Apostolidis

**Affiliations:** ^1^Department of Chemistry and Food Science, Framingham State University, Framingham, MA, United States; ^2^U.S. Department of Agriculture, Agricultural Research Service, Albany, CA, United States

**Keywords:** witch hazel extract, hamamelitannin, *Lactobacillus plantarum*, prebiotic, oxidative stress protection

## Abstract

Witch hazel extract has been evaluated in prior studies demonstrating the phenolic-mediated biofilm inhibition, toxin production inhibition, and growth inhibition in *Staphylococcus aureus*. In this study, we are evaluating the possible prebiotic and protective effect of witch hazel extract on select probiotic *Lactiplantibacillus plantarum* strains, namely *L. plantarum* LP 10241 and *L. plantarum* LPBAA-793. When the prebiotic effect was evaluated, we observed that the tested extract had prebiotic effect at the higher tested dose (0.5%) on LPBAA-793 strain (8.7 log CFU/mL after 18 h compared to 5.1 log CFU/mL with the control) and on LP 10241 strain (7.7 log CFU/mL after 18 h compared to 4.4 log CFU/mL with the control). For the evaluation of the protective effect of witch hazel extract on the select strains, we subjected nutrient depletion stress under aerobic conditions and monitored the cell death with and without addition of witch hazel extract. We observed that the tested extract had a significant protective effect on LPBAA-793 strain (4 log CFU/mL after 12 days, compared to no growth with control) and a slighter protective effect against LP 10241 strains (6.3 log CFU/mL in day 2 compared to 4.3 log CFU/mL with control). The results from this research provide for the first time the rationale that while witch hazel extract has significant antimicrobial, anti-toxin production and anti-biofilm activities on pathogenic microorganisms, it might play an important and positive role on health-beneficial probiotic bacteria.

## Introduction

Gut bacteria affect health in multiple ways including immune functions and metabolism. A rich and diverse gut microbiota is considered as promoting health while preventing chronic diseases. In contrast, poor diversity of the gut ecosystem is characteristic of chronic diseases, including obesity, diabetes, asthma, and gut inflammatory disorders (1). Due to the general bacterial-killing nature of antibiotics, repetitive use of antibiotics deprives people of a rich gut bacterial environment and this leads to adverse health effects. Antibiotic use can lead to disruption of the normal microflora, potentially giving rise to other health issues, like the rise in secondary *Clostridium difficile* infections causing antibiotic-associated diarrhea. According to the Center for Disease Control, *C. difficile* has become the most common microbial cause of hospital acquired infections in U.S. hospitals, resulting in thousands of deaths and $4.8 billion each year in excess health care costs for acute care facilities alone (2).

It has also been shown that while the gut microbiome of healthy adults is resilient and able to recover after short-term simultaneous exposure to three different antibiotics, the exposure to broad-spectrum antibiotics may reduce the diversity of the intestinal bacterial ecosystem (3). In fact, populations in the developed world have a considerably lower diversity of their gut microbiota than native people living in certain parts of Africa and Amazonas. One possible explanation for this may be the widespread use of antibiotics in the treatment of infectious diseases (4).

We used a witch hazel extract (WH) that contains a Hamamelitannin (HAMA), that is known to inhibit biofilm formation and toxin production (5–7). We showed that WH had growth inhibitory effects both on gram positive and gram-negative bacteria, with varying efficacies (0.05–10% WH). This suggests that efficacy may depend more on the molecular mechanisms of suppression involved, like interference with quorum sensing by HAMA (8) and/or disruption of cell membrane function by gallic acid (9). The most WH-sensitive ones tested were *Staphylococci* (including MRSA) and Enterococci, followed by *Acinetobacter, Klebsiella, Escherichia, Pseudomonas*, and streptococci. Importantly, resistance to WH has not been demonstrated and WH is effective also against antibiotic resistant strains.

Witch hazel was shown to inhibit biofilm formation and toxin production. Importantly, toxin inhibition was also evident in the presence of sub-inhibitory concentrations of the antibiotic ciprofloxacin that actually induces toxin production at these low concentrations (6). This suggests that WH can be used as an additive or alternative to antibiotics, to inhibit bacterial growth and pathogenesis, thus reducing the need for using excessive amounts of commonly used antibiotics and potentially in preventing bacteria from producing the many types of toxins.

Additionally, phenolic phytochemicals, including compounds similar to HAMA, have been shown to be metabolized by gut-microorganisms, without having the antimicrobial effect that has been widely demonstrated against pathogenic microorganisms (10). As a matter of fact, it has been demonstrated that certain polyphenols can have a prebiotic effect on select probiotics (11). More specifically for *Lactobacillus* strains prebiotic effect was observed with white and red wine phenolic-rich by-products (12), with pomegranate polyphenols (13) and with holy-basil, pepper and ginger (14). The observed prebiotic effect was attributed to the ability of *Lactobacillus* strains to metabolize polyphenols present in the tested extracts (12–14).

We hypothesize that the variability in sensitivity to WH can be beneficial in targeting bacterial infections, while not affecting normal gut microflora. The maintenance of normal gut microflora is critical for various health aspects. We have established that WH has excellent anti-toxin and anti-biofilm effects against enteric pathogens, but do not know if it can also act as a prebiotic, to help maintain healthy microbial microflora. Some natural products that have antimicrobial effects can also act as prebiotics, since the antimicrobial polyphenols they contain are metabolized only by specific probiotic bacterial strains. The aim of our research is to examine the effect of WH on gut microflora and thus determine if WH has a potential prebiotic effect. More specifically, in this research we are testing the effect of WH on probiotic bacteria belonging in the genus of *Lactobacillus* (two strains of *Lactobacillus plantarum*) (15, 16) and the potential preventive effect of WH on the same two *Lactobacillus plantarum* strains, following nutrient-depletion stress under aerobic conditions.

## Materials and Methods

### Bacteria

*Lactobacillus plantarum* ATCC 10241 (LP10241) and *L. plantarum* ATCC BAA-793 (LP 793) were used in this study. Both strains were received by ATCC as freeze-dried pellets, were reconstituted in MRS broth and incubated overnight anaerobically at 37°C. Then 100 μl from the overnight growth were inoculated in 10 mL MRS broth and incubated anaerobically overnight. Frozen stocks were prepared using 12% Non-fat dry milk and 2% glycerol solution and stored at −60°C.

### Witch Hazel Extract and Chemicals Used

whISOBAX witch hazel extract (WH) was supplied by StaphOff Biotech Inc., (Hopkinton, MA, United States). WH is a witch hazel extract in 50% ethanol. Unless noted, all chemicals were purchased from Sigma-Aldrich Co., (St. Louis, MO, United States).

### Total Phenolic Content

The total phenolic content was determined as described by Kang et al. (17), with modifications. Briefly, 0.2 mL of the sample (WH, GT, HAMA, or increasing concentrations of Gallic Acid standard) was mixed with 1 mL distilled water, 0.2 mL 95% ethanol and 0.1 mL 50% (v/v) Folin–Ciocalteau’s reagent, and incubated at 22°C for 5 min. One milliliter of 5% Na_2_CO_3_ was added, and the mixtures were kept in the dark at 22°C for 1 h. The solution was mixed by vortexing, and the absorbance was determined by measuring the absorbance at 725 nm using a 96-well plate. The results were expressed as mg of gallic acid equivalents (GAE) per gram of sample of dried extract weight (DW) or per sample volume. The data presented are an average of three measurements.

### Hamamelitannin Content in Witch Hazel (High Pressure Liquid Chromatography Determination)

Witch hazel was analyzed by High Pressure Liquid Chromatography (HPLC) and the HAMA content was determined by comparison to a standard HAMA sample, according to Wang et al. (18) with some modifications to provide a faster method that is less susceptible to solvent composition, and is compatible with LC requirements. The column used was the Durashell reverse phase C18 (Agilent Technologies, Santa Clara, CA, United States) 3 μm, 100 Ǻ, 4.6 × 50 mm column. The solvents used were acetonitrile/water (both containing 0.1% TFA) gradient. HPLC (Agilent 1200 System, Agilent Technology, Santa Clara, CA, United States) was used with a variable wavelength detector. The presence and amount of HAMA in WH was confirmed by comparing the retention time and absorbance spectrum with the HAMA standard and by using a HAMA standard curve.

### Prebiotic Effect Evaluation

The prebiotic effect was tested with both LP10241 and LP793 with various doses of witch hazel (WH) extract. The growth patterns of both LP10241 and LP793 were tested over an 18-h period. The cultures were inoculated from a frozen stock (100 μl) in MRS broth (10 mL) and incubated for 18-h at 37°C anaerobically. After 18-h incubation, a fresh overnight culture was prepared with another 18 h anaerobic incubation at 37°C. Then the cultures were diluted in MRS to a log CFU/mL around 3. From this dilution, the samples were inoculated in MRS broth for the control, and broth plus a dose of a WH extract. The extracts were prepared by adding the appropriate dose to 10 mL of MRS broth and autoclaving the media and extract in test tubes. Controls were prepared with varying doses of 50% ethanol as it is present in the extract itself, to evaluate the possible ethanol toxicity effect due to the extraction solvent used in the preparation of the WH extract. After inoculation, sampling was performed every 6 h (time 0, 6, 12, 18), diluted and then plated in MRS agar. After 24 h incubation at 37°C using anaerobic conditions, the plates were read for quantification of lactic acid bacteria over time. The growth curves of the controls versus the WH extract were compared to understand if there is a prebiotic effect for either strain.

### Evaluation of Protective Effect Under Aerobic Conditions and Nutrient-Depletion Stress

Then we evaluated the protective effect of WH on the same strains, when the strains are subjected to aerobic conditions and nutrient-depletion stress. Both strains were inoculated (100 μL) in MRS broth and incubated at 37°C for 18 h. Growth test tubes were centrifuged for 10 min, and the cell pellet was resuspended in 10 mL 0.1% peptone water. This process was repeated three times; on the final resuspension the broth and botanicals previously prepared according to correct dose was used to suspend the pellet. The WH treatment was prepared by adding the appropriate dose in 10 mL of 0.1% peptone water and then autoclaved and used to resuspend the cell pellets. The extracts were tested in triplicates for each trial. The samples were then incubated aerobically at 37°C. Samples were plated at day 0, 1, 2, 3, 6, 9, and 12.

### Statistical Analysis

Experiments were done in triplicates and their averages presented. Means, standard errors, standard deviations and degree of significance (using ANOVA) were calculated from replicates within the experiments and analyses were done using Microsoft Excel XP.

## Results

### Total Phenolic Content and Phenolic Profile of Witch Hazel Extract

**FIGURE 1:**
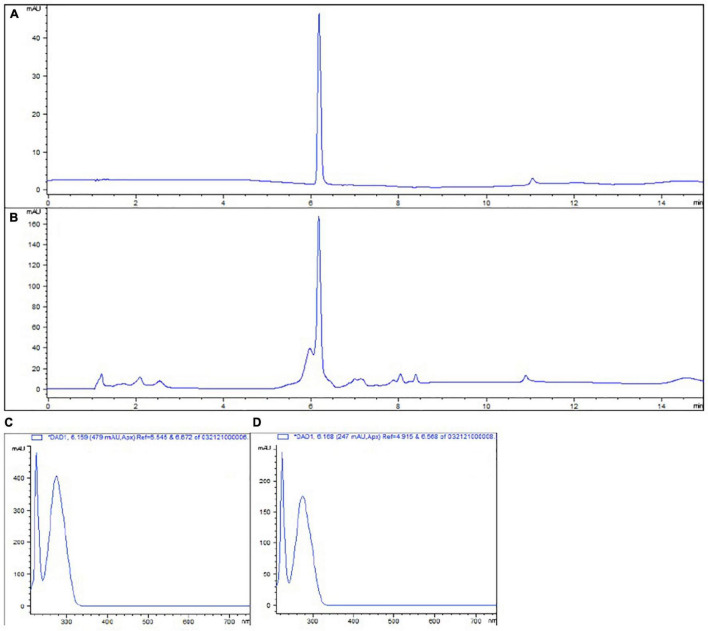
High Pressure Liquid Chromatography (HPLC) chromatogram of HAMA standard **(A)** and WH extract **(B)** at 280 nm. Absorbance spectrum of HAMA standard **(C)** and compound eluted around 6 min with WH extract **(D)**. HAMA elutes around 6 min for both the standard **(A)** and the WH extract **(B)**. Identification happens by comparing the absorbance spectrums of this peak both in the standard **(C)** and the WH extract **(D)**.

The total phenolic content and phenolic profile of the tested extract were evaluated as described in the materials and methods. The total phenolic content was determined to be 24.80 mg/mL GAE. When the HPLC profile analysis was performed we determined that the HAMA content in the tested extract was 15.35 mg/mL. HAMA in the extract was identified using a standard and comparing the corresponding absorbance spectrum (Figure 1).

### Prebiotic Effect of Witch Hazel Extract

**FIGURE 2:**
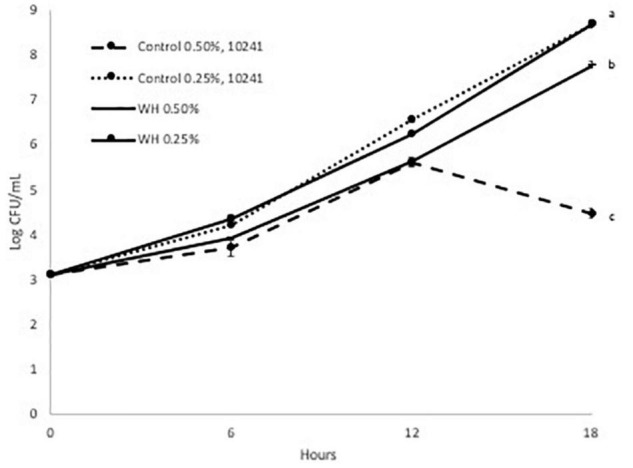
Growth curve of LP 10241 at 37°C with and without WH extract (a, b, c: values with the same letter are not significant different at *p* < 0.05).

**FIGURE 3:**
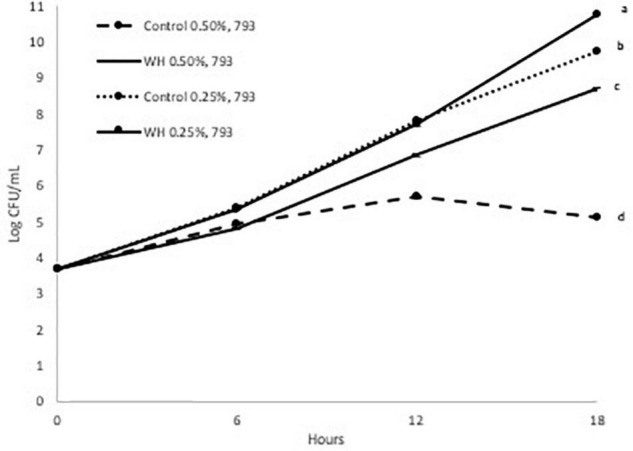
Growth curve of LP 793 at 37°C with and without WH extract (a, b, c, d: values with the same letter are not significant different at *p* < 0.05).

We evaluated the prebiotic effect of WH extract on the two *L. plantarum* strains at two doses (0.25 and 0.5%). Since WH extract was extracted using ethanol, the equivalent amount of ethanol was added in both controls. Initially, we observed that the growth of both probiotic strains was adversely affected from the presence of ethanol only at the 0.5% dose (Figures 2, 3).

When the LP10241 strain was tested, we observed that there was no effect of 0.25% WH extract on the growth curve for 18 h (Figure 2). When the 0.5% dose of WH extract was evaluated with the same strain, we observed that the growth of the control was affected (4.4 log CFU/mL after 18 h), however in the WH extract sample the LP10241 strain continued to grow without a problem (7.7 log CFU/mL after 18 h) (Figure 2).

When the LP793 strain was tested, we observed a slight prebiotic effect of 0.25% WH extract on the growth after 18 h (Figure 3). More specifically, addition of 0.25% WH extract resulted to a 1 log CFU/mL higher cell count after 18 h, compared to the control. When the 0.5% dose of WH extract was evaluated with the same strain, similarly to the previous strains, we observed that the growth of the control was affected (5.1 log CFU/mL after 18 h), however in the WH extract sample the LP793 strain continued to grow without a problem (8.7 log CFU/mL after 18 h) (Figure 3).

Based on the above, it is evident that WH extract had a protective effect, by significantly reducing the effects of ethanol toxicity that was observed at both strains at the 0.5% dose (Figures 2, 3). Additionally, 0.25% WH extract had a prebiotic effect on LP793 strain (Figure 3).

### Protective Effect of Witch Hazel Extract

Both tested strains were grown overnight and then the cells were collected and resuspended in 10 mL 0.1% peptone water, with and without 0.25% WH and incubated at 37°C aerobically. The 0.5% WH dose was not evaluated, due to the observed ethanol toxicity in the controls, since in this experiment we wanted to evaluate the protective effect of WH extract, resulting from nutrient depletion and aerobic conditions.

**FIGURE 4:**
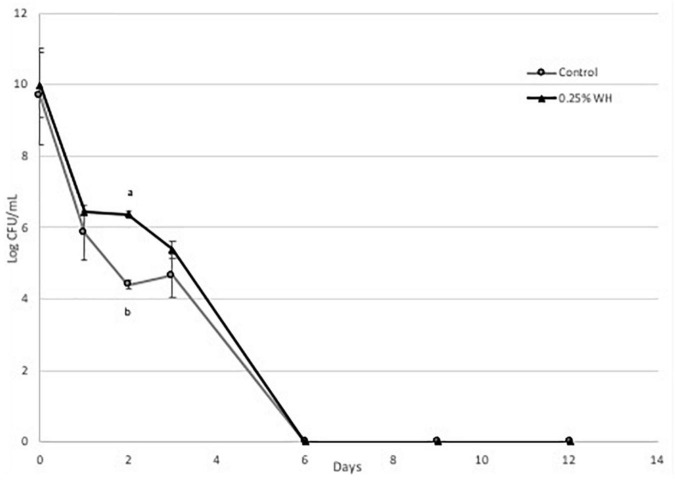
Death curve of LP 10241 at 37°C with and without 0.25% WH extract addition (a, b: values with the same letter are not significant different at *p* < 0.05).

**FIGURE 5:**
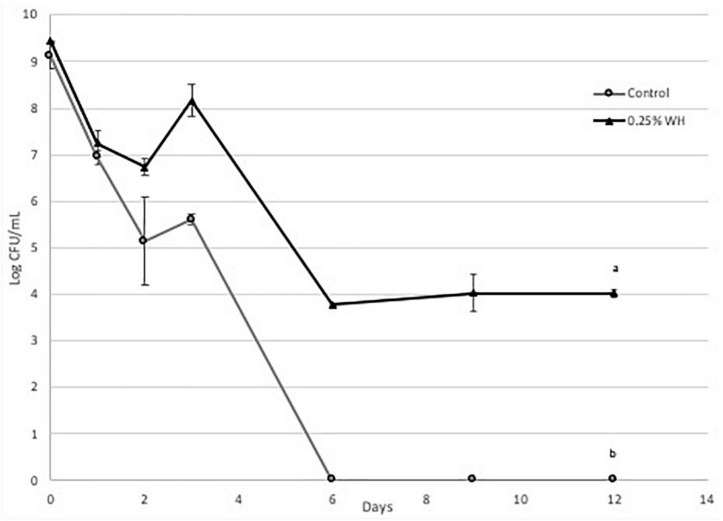
Death curve of LP 793 at 37°C with and without 0.25% WH extract addition (a, b: values with the same letter are not significant different at *p* < 0.05).

Briefly, we observed that WH extract had a protective effect at both strains at the tested dose (Figures 4, 5). More specifically, with strain LP 10241 we observed that in both control and treatment, viable cell growth was not detected at day 6 (Figure 4). However, the death rate of LP 10241 was lower with WH extract treatment. At day 2 the control had a 4.3 log CFU/mL growth while the WH sample had a 6.3 log CFU/mL growth (Figure 4). Also, at day 4 the control resulted to a 4.6 log CFU/mL growth, while the WH sample resulted to a 5.3 log CFU/mL count (Figure 4).

The protective effect of WH was significantly more pronounced with strain LP 793 (Figure 5). Similarly, to strain LP10241we observed a lower death rate. However, a very interesting observation with strain LP 793 was that the although the control resulted to no viable cells after day 6, the WH treatment maintained a steady cell growth until day 12 of 4 log CFU/mL (Figure 5).

Based on the above, we can state that WH extract at the tested dose has a protective effect to both strains against cell death, induced by starvation stress and aerobic growing conditions. This effect is significantly more pronounced with LP793 strain, since even after 12 days we could still detect 10^4^ CFU/mL living cells, while no living cells could be detected after day 6 with the control.

## Discussion

Previous reports have demonstrated that WH extract containing high levels of HAMA can have significant effects in controlling biofilm formation, toxin production and proliferation of certain pathogenic bacteria (5–7). More specifically, HAMA has been shown to increase antibiotic susceptibility of *Staphylococcus aureus* biofilms by affecting peptidoglycan biosynthesis and eDNA release (19). Additionally, HAMA from WH has demonstrated specific cytotoxic activity against colon cancer cells (20) and anti-TNF activity (21). The free radical scavenging activity of witch hazel HAMA compound was also observed in a trial concerning cancer cells. The scavenging capabilities were observed to be higher in the galloylated fractions (22). This information is relevant as these modified phenolic compounds proved to have higher antioxidant capabilities, which may be relevant to this research as these induced phenolics in fermentation were able to have a strong protective effect. However, the possible prebiotic effect of HAMA-containing WH has not been evaluated so far.

When we evaluated the possible prebiotic effect at two WH doses (0.5 and 0.25%) with two *L. plantarum* strains (LP10241 and LP793) we observed intriguing results. When tested at the lower dose (0.25%) no inhibitory effect was observed compared to control, but no clear prebiotic effect could also be suggested (Figures 2, 3). Only a slight prebiotic effect was observed with strain LP 793 (Figure 3). However, at the higher tested dose (0.5%) we observed that the ethanol present during the extraction had a negative effect on the growth of the control strains (Figures 2, 3). When WH extract was added at 0.5% dose, we observed that both strains had had significantly higher growth after 18 h (Figures 2, 3). Since it seems that addition of WH extract in both cases resulted to growth recovery, we can suggest that HAMA-containing WH extract protects the tested strains from ethanol-induced toxicity. Previous studies have demonstrated that ethanol exposure results to ROS accumulation that negatively affects metabolic and physiological cell processes (23, 24). The antioxidant and free-radical ability of polyphenols, such as HAMA, has been widely defined in various research efforts. We believe that the observed protective effect could be due to the radical scavenging ability of the tested WH extract.

Then we evaluated the possible protective effect of WH extract at the lower dose (0.25%), to avoid any effects of ethanol toxicity, on the same two strains, when subjected to aerobic conditions and nutrient-depletion stress. We observed that WH addition had a protective effect on both strains, however the effect on strain LP 793 was more pronounced, since living cells were identified until day 12 (Figures 4, 5).

Here we report for the first time the protective effect of HAMA-enriched WH extract on *L. plantarum* strains under aerobic conditions and nutrient depletion stress. Many lactic acid bacteria can grow in the presence of oxygen but this results to the generation and accumulation of ROS, that will eventually introduce an oxidative stress (25). When species belonging in the Lactobacillus family are introduced to oxidative and nutrient-depletion stress they switch to heterofermentation and heavily depend on NADH dehydrogenase to remove reactive oxygen species (26). Based on our observations, we can suspect that eventually NADH dehydrogenase is overwhelmed and the accumulation of ROS within the cytoplasm results to cell death. Phenolic phytochemicals are well defined scavengers of reactive oxygen species. We suspect that HAMA-enriched WH extract reduces the reactive oxygen species stress on the tested strains resulting to the observed protective effect. Previous research suggested a potential mechanism for the protective effect of HAMA as it is said to have reacted with superoxide radicals, which in turn allowed dermal fibroblast cells to survive ROS exposure (27). This was observed by the rapid disappearance after introduction of ROS. In our research with lactic acid bacteria, it is important to note that even though no viable cells were observed with both strains after 6 days, the effect of WH was more pronounced with LP 793 strain (Figure 5). This is a very interesting observation and could involve the unique physiology of the two strains. It would be interesting in the future to perform the same study using qPCR, since it is possible that in the LP 793 strain we the control cells entered the VBNC (viable but non-culturable) state.

## Conclusion

This is the first evaluation of WH extract on the possible prebiotic or protective effect on lactic acid bacteria. Our results suggest that although no clear prebiotic effect was observed, a significant protective effect resulted when the tested strains were subjected to oxidative and nutrient-depletion stress. Based on our observations, this effect could be strain dependent, but also it could be extract dependent. It is quite possible that different extracts with different phenolic compounds can lead to different effects. Further studies using evaluating specific metabolic changes are necessary to determine the possible mechanism of action for the observed effects.

## Data Availability Statement

The raw data supporting the conclusions of this article will be made available by the authors, without undue reservation.

## Author Contributions

MF performed the research and carried out the data analyses. JL performed the HPLC analysis. RR and EA designed the experiments, overlooked the execution, and prepared the original draft of the manuscript. All authors contributed to the article and approved the submitted version.

## Conflict of Interest

The authors declare that the research was conducted in the absence of any commercial or financial relationships that could be construed as a potential conflict of interest.

## Publisher’s Note

All claims expressed in this article are solely those of the authors and do not necessarily represent those of their affiliated organizations, or those of the publisher, the editors and the reviewers. Any product that may be evaluated in this article, or claim that may be made by its manufacturer, is not guaranteed or endorsed by the publisher.
